# Seasonal climatic instability in the western Chinese Loess Plateau during Marine Isotope Stages 12–10

**DOI:** 10.1038/s41598-023-32923-8

**Published:** 2023-04-07

**Authors:** Fengjiang Li, Naiqin Wu, Yajie Dong, Yiquan Yang, Yueting Zhang, Dan Zhang, Qingzhen Hao, Houyuan Lu

**Affiliations:** 1 Key Laboratory of Cenozoic Geology and Environment, Institute of Geology and Geophysics, Chinese Academy of Sciences, Beijing, 100029 China; 2 Innovation Academy for Earth Science, Chinese Academy of Sciences, Beijing, 100029 China; 3 CAS Center for Excellence in Life and Paleoenvironment, Beijing, 100044 China; 4 Gansu Civil Air Defence, Lanzhou, 730010 China; 5 College of Earth Sciences, Hebei GEO University, Shijiazhuang, 050031 China; 6 School of History, Renmin University of China, Beijing, 100872 China; 7 College of Earth and Planetary Sciences, University of Chinese Academy of Sciences, Beijing, 100049 China

**Keywords:** Climate sciences, Palaeoclimate

## Abstract

Because of similar astronomical background, Marine Isotope Stage (MIS) 11 is viewed as an analogue of the Holocene, but the evolution of seasonal climatic instability during MIS 11 has not been well investigated. Here we present a time series of land-snail eggs—a recently-developed proxy of seasonal cooling events—from the Chinese Loess Plateau (CLP) to investigate seasonal climatic instability during MIS 11 and adjacent glacials. Due to the impact of low temperatures on egg hatching, egg-abundance peaks document seasonal cooling events. A total of five egg-abundance peaks were recorded in the CLP during MIS 12, MIS 11 and MIS 10. Three peaks are strong and occur close to glacial inception or interglacial-to-glacial transition; two weaker peaks occur during MIS11. These peaks imply seasonal climatic instability intensifies mainly during glacial initiation or transition. All these events correspond to ice-sheet growth and the loss of ice-rafted debris at high northern latitudes. Moreover, they occurred at the minima of local spring insolation during the MIS 12 and MIS 10 glacials, but at the maxima during the MIS 11 interglacial. This may contribute to the difference in the intensity of seasonal cooling events between low-eccentricity glacials and interglacials. Our results provide new evidence for understanding low-eccentricity interglacial–glacial evolution.

## Introduction

Seasonal climatic instability is a feature of the Earth’s climate system and may cause mass extinctions on longer timescales^1^. To gain insight into the future trajectory of the current interglacial, it is of vital importance to understand seasonal climatic instability during astronomical analogues of the Holocene and compare it with that of adjacent glacials. Marine Isotope Stage (MIS) 11 is considered as an analogue of the Holocene because both interglacials are characterized by a minimum of the 400-kyr (not 100-kyr) eccentricity cycle^2^. Using climatic proxies such as oxygen isotope from marine deposits, grain size, magnetic susceptibility (MS) and land snails from terrestrial records, the strength, duration and millennial climatic instability during MIS 12–10 have been examined^2–5^. However, seasonal climatic instability during this unique period is poorly understood, since conventional climatic proxies are mostly unable to clearly document climatic events from specific seasons^3,5^.

Land-snail eggs have recently been demonstrated to be a proxy of seasonal cooling events, based on the biological principle that temperature is the most important factor impacting egg hatching^6^. Recent investigation of modern eggs in surface soils of the Chinese Loess Plateau (CLP) and East China has indicated that during the reproductive season (mainly spring) land-snail eggs are more abundant in the CLP (where seasonal cooling events are strong) than in East China (where cold spells are weak)^7^. The present climate over the CLP and East China is characterized by seasonal alternations of the East Asian winter and summer monsoon circulations^5^. Seasonal cooling events, such as cold spells, are frequent during spring. They generate temperature decreases of more than 8–10 °C, which results in low spring temperatures unfavorable for land-snail eggs to hatch^7^. Indeed, changes in the abundance of modern land-snail eggs correspond well to changes in spring minimum temperature, with high egg abundance corresponding to low spring temperature that is mainly caused by seasonal cooling events such as cold spells^7^. These studies allow us to use fossil land-snail eggs to decipher the evolution of seasonal cooling events in the geological past.

**Figure 1:**
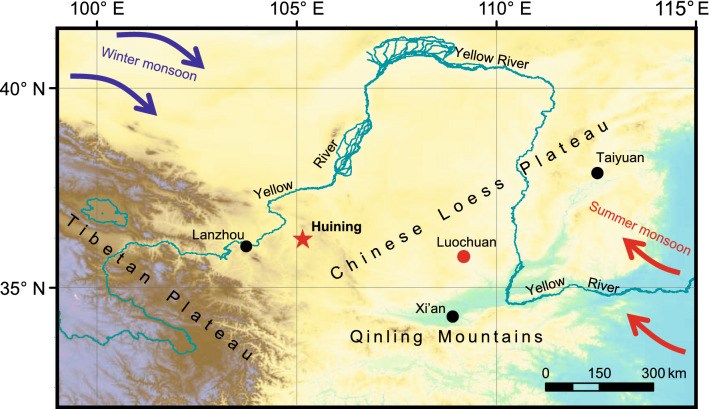
Locations of the Chinese Loess Plateau and the studied section. The red star denotes the location of the Huining loess–paleosol sequence. The black and red solid circles denote locations of major cities and the Luochuan section, respectively. The blue arrows indicate the direction of the East Asian winter monsoon, and the red arrows the East Asian summer monsoon. The base map of this figure was generated using DIVA-GIS 7.5 (http://www.diva-gis.org/).

Fossil land-snail eggs are abundant in loess-paleosol sequences of the CLP^8,9^. The loess (L) and paleosol (S) units are well correlated with even and odd stages of MIS record, respectively, which documents glacial-interglacial cycles^3,5,10,11^. The L5, S4 and L4 units provide one of the best terrestrial climatic records for MIS 12–10^5,10,11^. The Huining loess-paleosol sequence (36°14′N, 105°09′E) is located in the western CLP (Fig. 1) where paleosols are weakly developed^10^ and thus favor the preservation of land-snail eggs^9^. Here, seasonal cooling events were investigated for MIS 12–10, using well-preserved land-snail eggs from the Huining section.

## Results

### Land-snail eggs from the western CLP.

**Figure 2:**
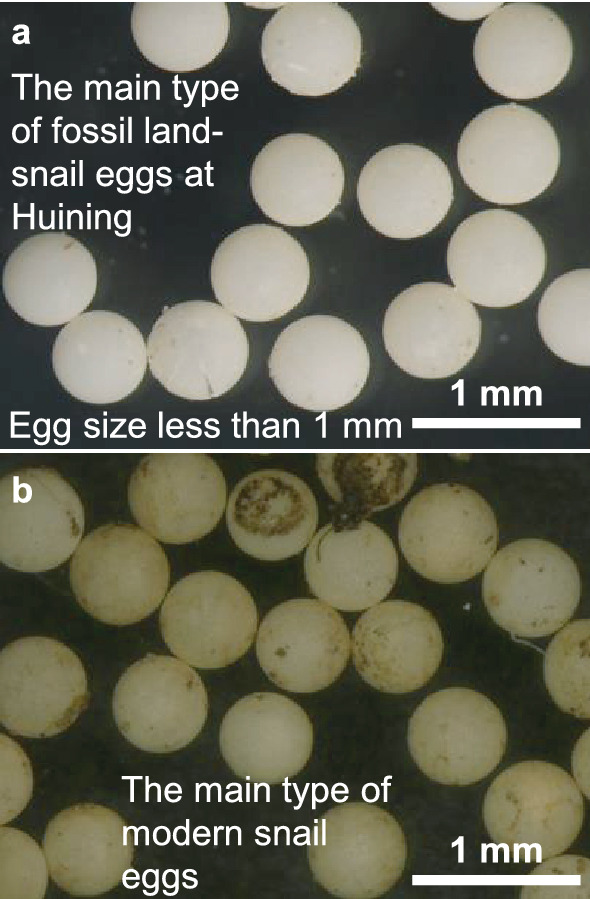
Fossil land-snail eggs from the Huining loess–paleosol sequence during MIS 12–10 and comparison with the same type of modern snail eggs from the Chinese Loess Plateau and East China. (**a**) Snail eggs from the Huining section. (**b**) The same type of modern snail eggs from surface soils of the Chinese Loess Plateau and East China.

**Figure 3:**
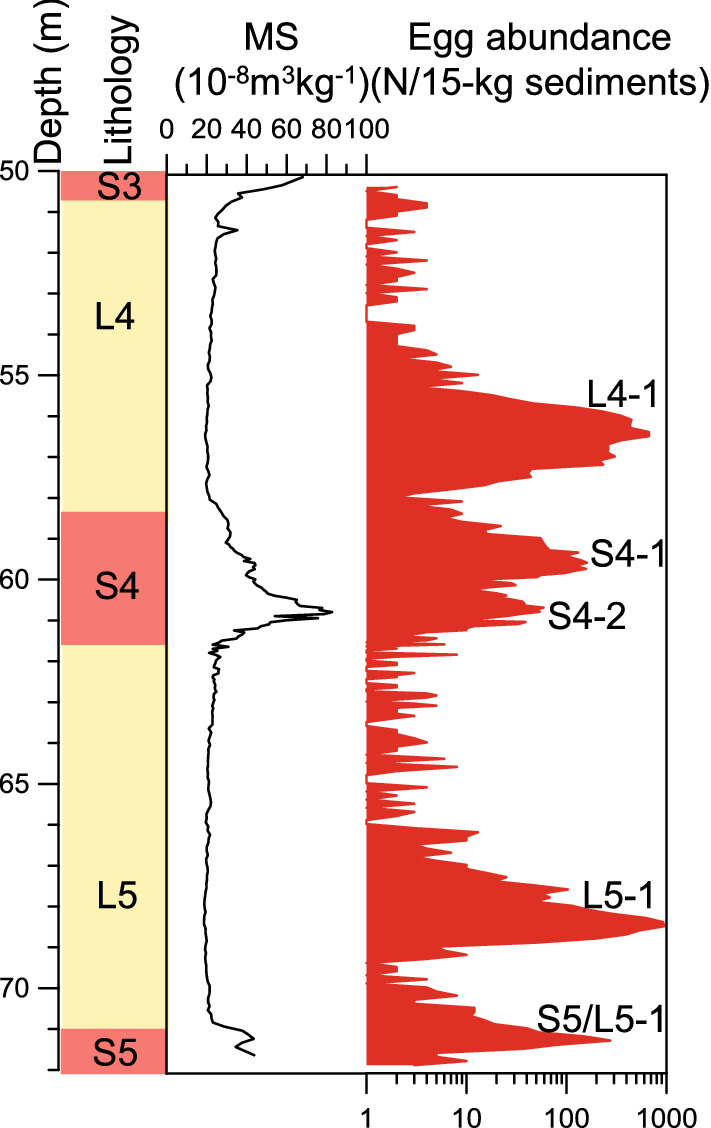
The depth, lithology, magnetic susceptibility (MS) and egg abundance from the L4, S4 and L5 units of the Huining loess–paleosol sequence. In the lithology column, the loess units (L4 and L5) are indicated by yellow shading and the paleosol units (the bottom of S3, entire S4 and upper S5) by red shading.

**Figure 4:**
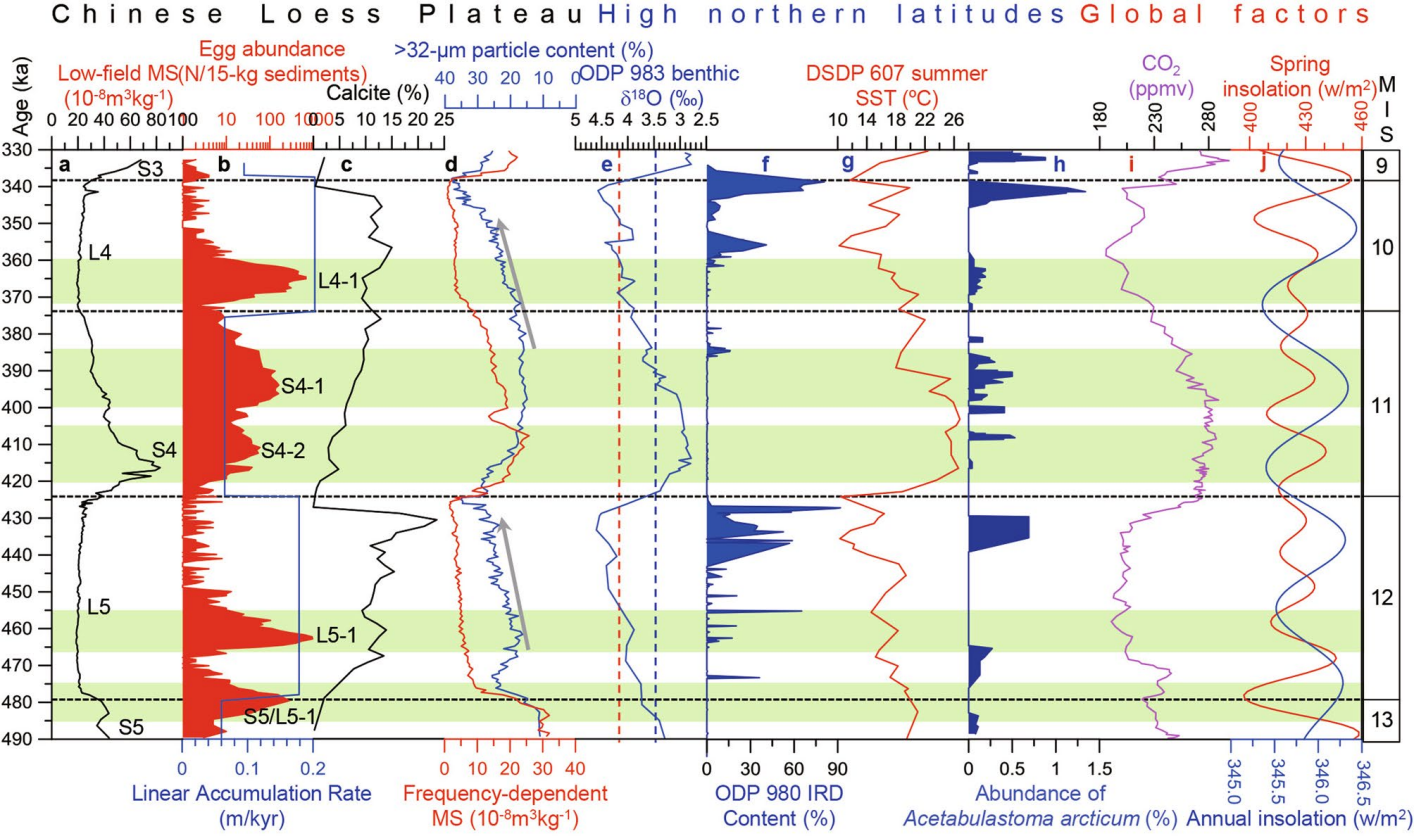
Seasonal cooling events in the Chinese Loess Plateau and their comparison with other records from the Chinese Loess Plateau (**a–d**), high northern latitudes (**e–h**) and global factors (**i,j**) during Marine Isotope Stages (MIS) 12–10. (**a,b**) Magnetic susceptibility (MS) record, seasonal cooling events (red) (L4-1, S4-1, S4-2, L5-1 and S5/L5-1, from top to bottom), as documented by egg-abundance peaks, and linear accumulation rate (blue) from the Huining section, respectively. The units of loess (L5 and L4) and paleosol (entire S4, and parts of S5 and S3) are labeled on the low-field MS record. (**c**) Calcite content in the Luochuan section^14^. (**d**) The content of coarse grain size (> 32 μm) (blue) and frequency-dependent MS (red) in the Luochuan section^5^. (**e**) Benthic δ^18^O record from Ocean Drilling Program (ODP) Site 983 located at 60.4° N, 23.64° W^16^. The red and blue dashed lines indicate the 4.2‰ and 3.5‰ thresholds of benthic δ^18^O values, respectively. (**f**) Ice-rafted debris (IRD) record from ODP Site 980 at 55° N, 15° W in the subpolar North Atlantic^17^. (**g**) Summer sea surface temperature (SST) record from Deep Sea Drilling Project (DSDP) Site 607 at 40° N, 32.97° W^18^. (**h**) Relative abundance of the ostracod *Acetabulastoma arcticum* which indicates sea ice cover in the Arctic Ocean^20^. (**i**) CO_2_ record from the European Program for Ice Coring in Antarctica (EPICA) Dome C ice core^19^. (**j**) Spring (red) and mean annual (blue) insolation at Huining located at the latitude of ~ 36º N^21^. MIS is labeled on the right.

Land-snail eggs with spherical or flat-spherical shape and yellow-white color (Fig. 2a) were retrieved from the L5, S4 and L4 units of the Huining section in the northwestern CLP. They were similar in size to those modern eggs from surface soils of the CLP and East China (Fig. 2b). Nearly all of the land-snail eggs were intact, indicating that they were unhatched (Fig. 2a). The largest dimension of the eggs is less than 1 mm but larger than 0.2 mm. Among all the 257 samples, 211 (over 80% of the total samples) yielded 13,580 eggs that were abundant at the transitional interval from S5 to L5 and the bottoms of L5 and L4, but less in S4 (Fig. 3, Supplementary Information). The maximum was 948 per 15-kg sediment which occurred at 68.4 m depth from the L5 loess unit (Fig. 3, Supplementary Information). The 46 samples that did not yield eggs occurred in the upper parts of loess units with very weak weathering as indicated by the low-field MS record and the content of carbonate (Fig. 4a,c).

### Seasonal cooling events documented by land-snail eggs

In this study, five seasonal cooling events were documented by land-snail eggs in the western CLP during MIS 12–10. Considering their occurrence in loess or paleosol units, we labeled them from top to bottom as L4-1, S4-1, S4-2, L5-1 and S5/L5-1. Their characteristics are shown in Fig. 4b and summarized in Table 1. They tended not to occur during warming transitions (deglacials) and glacial maxima over the entire time interval studied, but they intensified greatly during cooling transitions such as glacial inceptions and interglacial-to-glacial transitions. The strongest and second strongest events were L5-1 and L4-1, which occurred during glacial inceptions of MIS 12 and MIS 10, with the highest egg abundance being 948 and 668 per 15-kg sediment, respectively. They lasted for about 9 and 11 kyr. The third strongest was the transitional event S5/L5-1 which occurred during the transition from MIS 13 to MIS 12, with egg-abundance maximum being 273 per 15-kg sediment. It lasted for about 9 kyr. The three events mentioned above indicated that during MIS 12–10 seasonal climatic instability intensified when glacial initiated or was close to initiation, which is likely a new feature helpful for determining the initiation of glacials. The weakest and second weakest events S4-2 and S4-1 occurred during the low-eccentricity interglacial MIS 11, with their highest egg abundances only 58 and 159 per 15-kg sediment. All the five events are not documented by conventional climatic proxies, such as grain size, MS and benthic oxygen isotope^3,5^ (Fig. 4).

**Table 1:** Seasonal cooling events and their depth, age, duration and egg abundances in the Huining loess–paleosol sequence during MIS 12–10.

Seasonal cooling event	Depth (m)	Age (ka)	Loess (L)/paleosol (S)	Duration (kyr)	Intensity (maximum egg abundance in 15-kg sediment)
L4-1	55.4–57.6	360–371	L4	11	668
S4-1	58.9–59.85	385–399	S4	14	159
S4-2	60.25–61.15	406–419	S4	13	58
L5-1	67.1–68.8	456–465	L5	9	948
S5/L5-1	70.4–71.4	474–483	The boundary of S5/L5	9	273

## Discussion

Previous studies have shown abundant eggs in the surface soils of China and in the upper part of the Huining section^7,9^. After breaking some of these eggs, we observed embryonic land-snail shells inside the eggs^7,9^, which demonstrates undoubtedly that the eggs we observed are laid by land snails.

Egg cannibalism is unlikely in the Huining section. Egg cannibalism occurs when the eggs are laid as a clutch. Hatching asynchrony of the eggs in the clutch may cause the earliest hatchlings to eat the unhatched eggs within the same clutch^12^. However, the eggs in the Huining section are mainly from minute snails, as indicated by the size of the eggs (less than 1 mm in the largest dimension)^12,13^. Because minute snails mostly lay single eggs, i.e. not in a clutch^12,13^, egg cannibalism would not have been a dominant factor impacting egg abundance in the Huining section. Moreover, the intactness of nearly all eggs excludes significative influences of predator consumption of the eggs.

Changes in egg abundance could not have been significantly impacted by dust accumulation rate. Dust accumulated no more than 0.2 mm per year in the Huining section during the studied time interval (this study) (Fig. 4b). Egg hatching generally occurs on seasonal or yearly timescale^12,13^ and the sizes of the eggs we observed are larger than 0.2 mm and less than 1 mm (Fig. 2). Therefore, dust accumulation would not have a strong impact on egg hatching. This is consistent with the observation that changes in egg abundance do not parallel with linear accumulation rate in the Huining section (Fig. 4b).

Soil development is the main factor impacting carbonate content^4,5,14^ and therefore egg preservation^8,9^. In the CLP, high content of carbonate is generally associated with weak development of soils, and vice versa^4,14^. In the Huining section, changes in egg abundance seem not to have been impacted by carbonate dissolution for the following three reasons. First, higher content of carbonate in the Huining section favors the preservation of land-snail eggs. The average content of carbonate in bulk samples of the L4, S4 and L5 units of the Luochuan section in the southeastern CLP (Fig. 1) was 10%, with carbonate content in 80% samples greater than 5%—the proposed threshold of shell preservation (personal communication with Dr. Olivier Moine)—and the maximum being 24%^14^ (Fig. 4c). Because carbonate content increased from the southeastern to northwestern CLP^10,14^, the Huining section would have greater carbonate content than the Luochuan section, favoring egg preservations. Moreover, because of very weak development of soils, carbonate content in the Huining section is much higher than the threshold of eggshell dissolution^14^. Therefore, changing carbonate content down section would not have significantly affected the preservation of eggs and therefore changes in egg abundance in the Huining section. Second, the Huining loess-paleosol sequence is characterized by a very weak development of paleosols, as indicated by low-field MS record (Fig. 3). MS values in the L4, S4 and L5 units of the Huining section were between 18.7 and 82.7 × 10^−8^ m^3^ kg^−1^, with an average of 28.4 × 10^−8^ m^3^ kg^−1^ (Fig. 3). Specifically, in our studied section, MS values in the paleosol unit S4 ranged between 24.8 and 82.7, whereas the two loess units L4 and L5 showed MS values ranging from 19.2 to 37.7 and from 18.7 to 37.7, respectively (Fig. 3). These values were significantly lower than those from strongly-developed paleosols that were characterized by low-field MS values greater than 100–120 × 10^−8^ m^3^ kg^−14^. Third, eggs were abundant and nearly all of them were intact, indicating that they were well preserved.

Seasonal cooling events can abruptly cause low temperatures unfavorable for egg hatching^7^. Temperature is the most important factor influencing egg hatching of land snails^6–9,12,13^. Nearly all oviparous species have temperature thresholds for egg hatching^7^. When these thresholds are exceeded, abundant eggs fail to hatch^7–9,12,13^ and are likely preserved in surface soils. Indeed, surface-soil study has indicated that the highest abundance in modern land-snail eggs occurs in all the samples taken from the region with the strongest cold spells and the lowest spring minimum temperatures^7^. In the region with cold spells occurring at a moderate frequency, 80% of the samples yield eggs and the egg abundance is greatly reduced^7^. However, egg abundance is the lowest in the region with the weakest cold spells and the highest spring minimum temperatures^7^. Therefore, changes in egg abundance in the Huining section during MIS 12–10 would have documented seasonal cooling events. This is consistent with both biological principles^6^ and the studies of the past 350-kyr eggs^7,9^.

For better understanding the evolution of seasonal cooling events during MIS 12–10, we compare our results with other climatic records from the Chinese Loess Plateau, high northern latitudes and the globe (Fig. 4). The reasons for selecting the Luochuan section are as follows. First, the Luochuan section from the CLP is the most classic loess sequence of the globe and the time interval of MIS 12–10 of the section has been well studied^4,5,10,11,14^. Second, proxies from loess records of the CLP can be well correlated and therefore the Luochuan section can represent changes in the entire CLP at an orbital time scale^4,5,10,11^. The use of the ODP/DSDP sites from high northern latitudes is based on the following reasons. First, the climate of the CLP is closely related to changes in high northern latitudes^5,10^. Second, all the selected ODP/DSDP sites are classic sites documenting changes in high northern latitudes^15^, and data of the proxies selected are available^15–18,20^, covering the period of our study.

L5-1 and L4-1 occurred during the early parts of MIS 12 and MIS 10 (Fig. 4e), corresponding to glacial inceptions defined by the Past Interglacials Working Group of PAGES (2016)^15^. Frequency-dependent MS and coarse particle content at Luochuan (Fig. 1) showed gradually decreasing and increasing trends, respectively, indicating a cooling climate condition during these two periods^5^ (Fig. 4d). In the subpolar North Atlantic (Ocean Drilling Program (ODP) Site 983, 60.4°N), during L5-1 the benthic foraminiferal δ^18^O values continued an increasing trend and finally crossed the 4.2‰ threshold, indicating large-amplitude growth of ice sheets^16^ which was further supported by the occurrence of ice-rafted debris (IRD) at ODP Site 980^17^ (Fig. 4e,f). Similarly, during L4-1, the benthic δ^18^O values at ODP Site 983 remained around 4.1‰/4.2‰, indicating that the ice sheets could have remained sufficiently large, but their further growth was delayed^5^. Very few of IRD at ODP Site 980 during L4-1 (Fig. 4f) confirmed that the ice sheets could not have expanded to the shore of the North Atlantic^17^. Moreover, declined sea surface temperature (SST) at Deep Sea Drilling Project (DSDP) Site 607 (40° N) and low atmospheric CO_2_ concentrations (~ 200 ppmv) further supported cooling climate conditions during L5-1 and L4-1^18,19^ (Fig. 4g,i). Low sea ice would have also intensified L5-1 and L4-1^20^ (Fig. 4h). It should be pointed out that although uncertainty of hundreds to a few thousand years in our chronology may exist^3,5,15^, the comparison of the Huining record with most other records is based on the chronology of benthic foraminiferal δ^18^O stack (LR04)^3^. In other words, our comparison is mainly based on the same chronology^3^. Therefore, the comparison is unlikely to be significantly impacted by age uncertainty.

L5-1 and L4-1 corresponded to minima in local spring insolation, with the lowest value of 411 w/m^2^ corresponding to the strongest event L5-1 and a higher value of 420 w/m^2^ to the second strongest event L4-1^21^ (Fig. 4j). These insolation minima would be helpful for yielding lower temperatures during the reproductive season, spring^7,9^. These two events did not correspond well to minima in mean annual insolation (Fig. 4j), confirming that seasonal, rather than annual, cooling events are responsible for the occurence of egg-abundance peaks.

When L5-1 and L4-1 terminated, the benthic δ^18^O values at ODP Site 983 exceeded the 4.2‰ threshold of ice-sheet growth^16^, which indicated that ice sheets during the glacial maxima of MIS 12 and MIS 10 could have grown substantially to reach the shore of the subpolar North Atlantic, discharging abundant IRD-loaded icebergs into the ocean (Fig. 4f). No seasonal cooling events were observed under such conditions during MIS 12–MIS 10.

S5/L5-1 occurred during the transition from MIS 13 to MIS 12. Both coarse particle content and MS records from the CLP showed prominent changes, indicating a strong cooling^5^ (Fig. 4a,d). This period is characterized by benthic δ^18^O values exceeding the 3.5‰ threshold, but not reaching the 4.2‰ threshold, in the subpolar North Atlantic^16,17^ (Fig. 4e). This indicates strong growth of ice sheet at high northern latitudes, but the growth may not be greater than those during L5-1 and L4-1. Coincidently, decreases in summer sea surface temperature at DSDP Site 607 were smaller during S5/L5-1 than during L4-1^18^ (Fig. 4g). IRD was absent in the subpolar North Atlantic during this period^17^ (Fig. 4f); the Arctic Ocean was absent of sea ice cover, as indicated by the paucity of the sea-ice-related ostracod species *Acetabulastoma arcticum*^20^ (Fig. 4h). Atmospheric CO_2_ concentration decreased by 20 ppmv^19^ (Fig. 4i). Local spring-insolation minimum (397 w/m^2^), rather than mean annual insolation^21^ (Fig. 4j), would have favored the occurrence of low temperatures unsuitable for egg hatching during the reproductive season.

S4-1 and S4-2 occurred within the low-eccentricity interglacial MIS 11, but during the last three high-eccentricity interglacials they were rare (absent if considering only MIS 5e as the last interglacial)^9^. Their intensities are significantly lower than those of L4-1, L5-1 and S5/L5-1 (Fig. 4b, Table 1). The weakest event S4-2 occurred during the warmest interval of MIS 11^2,15^, as indicated by the highest MS values, the lowest benthic δ^18^O (2.79–3.2‰, not exceeding the 3.5‰ threshold), the absence of IRD, the paucity of sea ice, and the highest SST (> 25 °C) and atmospheric CO_2_ concentrations (265–287 ppmv)^5,16–20^ (Fig. 4d–i). However, S4-1 occurred during late MIS 11 that was indicated by numerous proxies to be cooling conditions^4,5,16,18–20^. The MS values in the CLP decreased^5^ (Fig. 4a,d). The benthic δ^18^O values in the subpolar North Atlantic were surpassing the threshold of 3.5‰, which documented ice-sheet growth by an increase of 0.7‰ in benthic δ^18^O^16^ (Fig. 4e). Such an amount of growth could not have caused the margin of the ice sheets to reach the North Atlantic because IRD was absent at ODP 980^17^ (Fig. 4f). Associated with ice-sheet growth was a strong decrease of ~ 8 °C in summer sea surface temperature^18^ (Fig. 4g). Sea ice cover in the Arctic Ocean expanded, as indicated by moderate abundance of *Acetabulastoma arcticum*^20^ (Fig. 4h). CO_2_ concentration decreased ~ 40 ppmv^19^ (Fig. 4i).

Different from those cooling events during glacials, S4-1 and S4-2 corresponded to peaks in spring insolation, which may have contributed to their weaker intensities. Moreover, higher spring-insolation maxima may induce weaker seasonal cooling events. For example, S4-2, with spring-insolation maximum of 441 w/m^2^, was weaker than S4-1 that has a lower maximum of 435 w/m^2^ (Fig. 4j)^21^.

Ice volume changes, including ice-sheet growth and sea ice retreat, would strengthen seasonal cooling events in the CLP^5,22^. Though strengthening the Siberian High, the growth of ice sheets (but not growing to the level of yielding IRD) would have enhanced seasonal cooling events (e.g., cold spells) in the CLP during glacial inceptions and interglacial-to-glacial transitions. However, during glacial maxima of the unique MIS 12–10, ice sheet would grow further to become sufficiently large for IRD-contained icebergs to discharge into the North Atlantic. This is likely to have resulted in a long, stable cooling climate conditions in spring in the CLP^23^, which were unfavorable for the occurrence of cooling events. Indeed, a previous study has indicated that climatic instability is likely to diminish when ice sheets are sufficiently large to surpass the ~ 4.2 to 4.6‰ threshold of the benthic δ^18^O values^17^. The lack of IRD imply probably that seasonal cooling events could have a mechanism different from the Heinrich events that were characterized by abundant IRD. This remains to be examined by simulations. Moreover, low sea ice cover in the Arctic Ocean would enhance seasonal cooling events in the CLP by strengthening the Siberian High and also by promoting the ocean–atmosphere heat/moisture exchange. This favors air movements from high northern latitudes to the CLP^24^, inducing strong cold spells. Cold spells generate temperature decreases over 8–10 °C^22^, which would exceed the lowest temperature required for land-snail egg to hatch and therefore lead to substantial hatching failures^7^.

Insolation is likely to favor the occurrence of seasonal cooling events through its impact on temperature. However, not all of the spring-insolation minima at 36° N induced seasonal cooling events. There were two spring-insolation minima within each glacial and interglacial, but seasonal cooling events occurred only at the earlier minimum of local spring insolation during MIS 12 and MIS 10. During MIS 11, they did not occur at any spring-insolation minimum, but rather at spring-insolation maxima.

## Conclusion

We present a time series of land-snail eggs from the weakly-weathered Huining loess-paleosol sequence in the western CLP to investigate seasonal climatic instability during the interglacial MIS 11 and adjacent glacials MIS 12 and MIS 10. Five seasonal cooling events, labeled from MIS 10 to MIS 12 as L4-1, S4-1, S4-2, L5-1 and S5/L5-1, were documented by peaks of land-snail egg abundance. They were strong during cooling transitions such as glacial inceptions and interglacial-to-glacial transitions. They also occurred, although weakly, during the low-eccentricity interglacial MIS 11, which differs greatly from high-eccentricity interglacials of the last 350 kyr^9^. The glacial-inception and transitional events L4-1, L5-1 and S5/L5-1 were the strongest, implying that during MIS 12–10 seasonal climatic instability intensified when glacial initiated or was close to initiation. Nearly all these events corresponded to the growth of ice sheets, with the benthic δ^18^O values not exceeding ~ 4.2‰ in the subpolar North Atlantic, and the loss of IRD. Moreover, seasonal cooling events occurred at the minima of local spring insolation during the MIS 12 and MIS 10 glacials, but at the maxima during the MIS 11 interglacial. This may contribute to stronger intensities of seasonal cooling events during glacials than those during interglacials. Our results suggest that the occurrence of seasonal cooling events is likely to be a new feature of glacial initiation, which may gain insights into the evolution of low-eccentricity interglacials and glacials. Since fossil land-snail eggs have not been extensively investigated around the CLP and the globe, future studies should focus on more sections from more regions and investigate spatial changes in land-snail egg abundance and seasonal cooling events.

## Methods

A total of 257 sediment samples were continuously taken from the Huining section. 10-cm sampling interval was used for the units from the top of S5 to the middle L5 and from L4 to the bottom of S3; 5-cm interval was used for the main part of S4 and upper part of L5 (Supplementary Information). Each sample weighed about 15 kg, corresponding in volume to ~ 15 L. Using a mesh of 0.5-mm diameter, we washed and sieved the sediment samples in the field and laboratory to concentrate fossil land snails and their eggs. Eggs were sorted, counted and measured under a Leica S9i microscope. The shells were also picked and identified under a Leica S9i microscope. They were counted using the extensively used protocols^4^. References used for species identification are the specimen stored in the Institute of Geology and Geophysics, Chinese Academy of Sciences, and the monographs by Yen (1939)^25^ and Chen and Gao (1987)^26^. Moreover, parallel with the egg samples, 257 powder samples were taken for the measurement of MS. The powder samples were air-dried in the laboratory, and then we used a Bartington MS meter to measure low-field MS values.

Using age controls from paleomagnetic boundaries^10^ and correlation between MS and MIS records^3,5^, the chronologies of Chinese loess established by different age models, such as orbital tuning, grain size and magnetic susceptibility, are consistent, with the upper part being confirmed by OSL and ^14^C ages^4,5,9,11,14,23^. Therefore, we established the timescale of the Huining section using linear interpolation between age controls yielded by correlating MS and MIS records^3,5^.

## Supplementary Information

Supplementary Information.

## Data Availability

The data gained by this work can be found in the supporting information.

## References

[1] Ivany LC, Patterson WP, Lohmann KC (2000). Cooler winters as a possible cause of mass extinctions at the Eocene/Oligocene boundary. Nature.

[2] Candy I, Schreve DC, Sherriff J, Tye GJ (2014). Marine Isotope Stage 11: Palaeoclimates, palaeoenvironments and its role as an analogue for the current interglacial. Earth-Sci. Rev..

[3] Lisiecki LE, Raymo ME (2005). A Pliocene-Pleistocene stack of 57 globally distributed benthic δ^18^O records. Paleoceanography.

[4] Wu NQ (2007). Climatic conditions recorded by terrestrial mollusc assemblages in the Chinese Loess Plateau during marine Oxygen Isotope Stages 12–10. Quat. Sci. Rev..

[5] Hao QZ (2012). Delayed build-up of Arctic ice sheets during 400000-year minima in insolation variability. Nature.

[6] Brown JH, Gillooly JF, Allen AP, Savage VM, West GB (2004). Toward a metabolic theory of ecology. Ecology.

[7] Li FJ (2021). Land-snail eggs as a proxy of abrupt climatic cooling events during the reproductive season. Sci. Bull..

[8] Li FJ, Yang YQ, Wu NQ, Huang LP, Dong YJ (2019). Fossil snail eggs discovered from the Chinese Loess Plateau and their indications of seasonal abrupt climate event. Quat. Sci..

[9] Li FJ (2022). Glacial-interglacial evolution of seasonal cooling events documented by land-snail eggs from Chinese Loess. Quat. Sci. Rev..

[10] Liu T (1985). Loess and the Environment.

[11] Kukla G (1987). Loess stratigraphy in central China. Quat. Sci. Rev..

[12] Barker GM (2001). The Biology of Terrestrial Molluscs.

[13] Welter-Schultes F (2012). European Non-Marine Molluscs, a Guide for Species Identification.

[14] Meng XQ (2018). Mineralogical evidence of reduced East Asian summer monsoon rainfall on the Chinese Loess Plateau during the early Pleistocene interglacials. Earth Planet. Sci. Lett..

[15] Past Interglacials Working Group of PAGES (2016). Interglacials of the last 800000 years. Rev. Geophys..

[16] Raymo ME (2004). Stability of North Atlantic water masses in face of pronounced climate variability during the Pleistocene. Paleoceanography.

[17] McManus JF, Oppo DW, Cullen JL (1999). A 0.5-million-year record of millennial-scale climate variability in the North Atlantic. Science.

[18] Ruddiman WF, Raymo ME, Martinson DG, Clement BM, Backman J (1989). Pleistocene evolution: Northern Hemisphere ice sheet and North Atlantic ocean. Paleoceanography.

[19] Lüthi D (2008). High-resolution carbon dioxide concentration record 650,000–800,000 years before present. Nature.

[20] Cronin TM (2019). Interglacial paleoclimate in the Arctic. Paleoceanogr. Paleocl..

[21] Laskar J (2004). A long-term numerical solution for the insolation quantities of the Earth. Astron. Astrophys..

[22] Qian LQ (1991). Climate of Loess Plateau.

[23] Huang LP, Wu NQ, Gu ZY, Chen XY (2012). Variability of snail growing season at the Chinese Loess Plateau during the last 75 ka. Chin. Sci. Bull..

[24] Wu BY, Su JZ, Zhang RH (2011). Effects of autumn-winter Arctic Sea ice on winter Siberian High. Chin. Sci. Bull..

[25] Yen TC (1939). Die Chinesischen land-und Süsswasser Gastropoden des naturmuseums senckenberg. Abh. Senckenberg. Nat. Ges..

[26] Chen DN, Gao JX (1987). Economic Fauna Sinica of China, Terrestrial Mollusca.

